# The effect of mechanosensitive channel MscL expression in cancer cells on 3D confined migration

**DOI:** 10.1063/1.5019770

**Published:** 2018-06-08

**Authors:** Johanna Heureaux-Torres, Kathryn E. Luker, Henry Haley, Matthew Pirone, Lap Man Lee, Yoani Herrera, Gary D. Luker, Allen P. Liu

**Affiliations:** 1 Department of Mechanical Engineering, University of Michigan, Ann Arbor, Michigan 48109, USA; 2 Department of Radiology, Center for Molecular Imaging, University of Michigan, Ann Arbor, Michigan 48109, USA; 3 Department of Biomedical Engineering, University of Michigan, Ann Arbor, Michigan 48109, USA; 4 Department of Immunology and Microbiology, University of Michigan, Ann Arbor, Michigan 48109, USA; 5 Cellular and Molecular Biology Program, University of Michigan, Ann Arbor, Michigan 48109, USA; 6 Biophysics Program, University of Michigan, Ann Arbor, Michigan 48109, USA

## Abstract

Metastatic cancer cells migrate through constricted spaces and experience significant compressive stress, but mechanisms enabling migration in confined geometries remain unclear. Cancer cell migration within confined 3-dimensional (3D) microfluidic channels has been shown to be distinct from 2D cell migration. However, whether 3D confined migration can be manipulated by mechanosensory components has not been examined in detail. In this work, we exogenously introduced a mechanosensitive channel of large conductance (MscL) into metastatic breast cancer cells MDA-MB-231. We discovered that inducing expression of a gain-of-function G22S mutant of MscL in MDA-MB-231 cells significantly reduced spontaneous lung metastasis without affecting the growth of orthotopic tumor implants. To further investigate the effects of G22S MscL on cell migration, we designed a microfluidic device with channels of various cross-sections ranging from a 2D planar environment to narrow 3D constrictions. Both MscL G22S and control breast cancer cells migrated progressively slower in more constricted environments. Migration of cells expressing MscL G22S did not differ from control cells, even though MscL was activated in cells in constricted channels of 3 *μ*m width. Interestingly, we found MscL expressing cells to be more frequently “stuck” at the entrance of the 3 *μ*m channels and failed to migrate into the microchannel. Our work demonstrates the possibility of engineering mechanotransduction for controlling confined cell migration.

## INTRODUCTION

Cancer metastasis involves extreme physical deformation of tumor cells with cross-sectional areas of 80–300 *μ*m^2^ through narrow paths and pores with cross-sectional areas of ≤60 *μ*m^2^.^1–3^ Such extreme deformation occurs mainly during intravasation and extravasation steps of the metastatic cascade and migration through a dense interstitial matrix.^4,5^ While these harsh physical barriers contribute to the inefficiency of the metastatic process,^6^ sufficient numbers of cancer cells complete the metastatic cascade to cause deaths of an estimated 90% of patients with solid tumors.^5,7,8^

Since cancer cell migration represents an essential step in metastasis, understanding mechanisms driving this process is essential to ultimately treating or preventing metastatic disease. Most studies of cancer cell migration rely on 2D environments with no spatial constraints. Under these conditions, migration depends on actin polymerization and polarization, Rho/ROCK myosin II contractility, integrin/matrix adhesion, and protrusion of lamellipodia type structures of the cell.^9^ However, 2D, unconstrained environments do not reproduce conditions *in vivo*, leading researchers to develop experimental platforms that better mimic architectural features cancer cells encounter *in vivo*. As one example of how studies in more physiologic environments reveal novel mechanisms of migration, recent work demonstrates that directional water permeation across a cancer cell drives migration within confined 3-dimensional (3D) microfluidic channels.^10^ Overexpression of an aquaporin, AQ5, and sodium/hydrogen exchangers at the cell membrane and their polarized distribution in cancer cells results in an “osmotic engine” propulsion system in which cancer cells have a net influx of water at the leading edge and net outflux of water at the trailing edge. This mode of migration occurs under extreme confinement (cross-sectional area of 30 *μ*m^2^) and is independent of cytoskeletal components.

Previous work established that a bacterial channel protein, mechanosensitive channel of large conductance (MscL), can be functionally expressed in mammalian cells.^11,12^ MscL opens in response to membrane tension and deformation with a gating threshold of ∼10.4 mN m^−1^. A gain-of-function mutant, MscL G22S, opens with a reduced force of ∼5–6 mN m^−1^ (Refs. 13–16) and can be similarly activated when reconstituted in mammalian cells.^17^ MscL has a large ∼3 nm diameter pore and functions as a non-selective channel, allowing bidirectional passage of any large osmolytes ≤10 000 Da in size.^18–20^ Mechanically activated ion channels in mammalian cells mediate a range of mechanosensitive responses,^21^ and the Piezo1 ion channel was shown to activate during 3D confined migration.^22^ We propose that extreme deformation of cancer cells during metastasis, in particular migration through narrow 3D confinements, will activate MscL at the plasma membrane and could potentially influence confined cell migration.

Here, we test the hypothesis that activation of MscL as cancer cells traverse confined spaces will disrupt and reduce metastasis. To investigate MscL's effect on cancer metastasis, we developed MDA-MB-231 human breast cancer cells, a highly metastatic cell line, stably expressing gain-of-function mutant MscL G22S. We examined the effects of MscL on primary tumor growth and spontaneous metastasis in an immunodeficient mouse model. Based on data showing the effects of G22S MscL on lung metastasis, we further investigated cancer cell migration in various microfluidic channel cross-sections to mimic different degrees of confinement *in vitro*.

## RESULTS

### Expression of the non-native channel, MscL G22S, impairs metastasis to the mouse lung

**FIG. 1.**
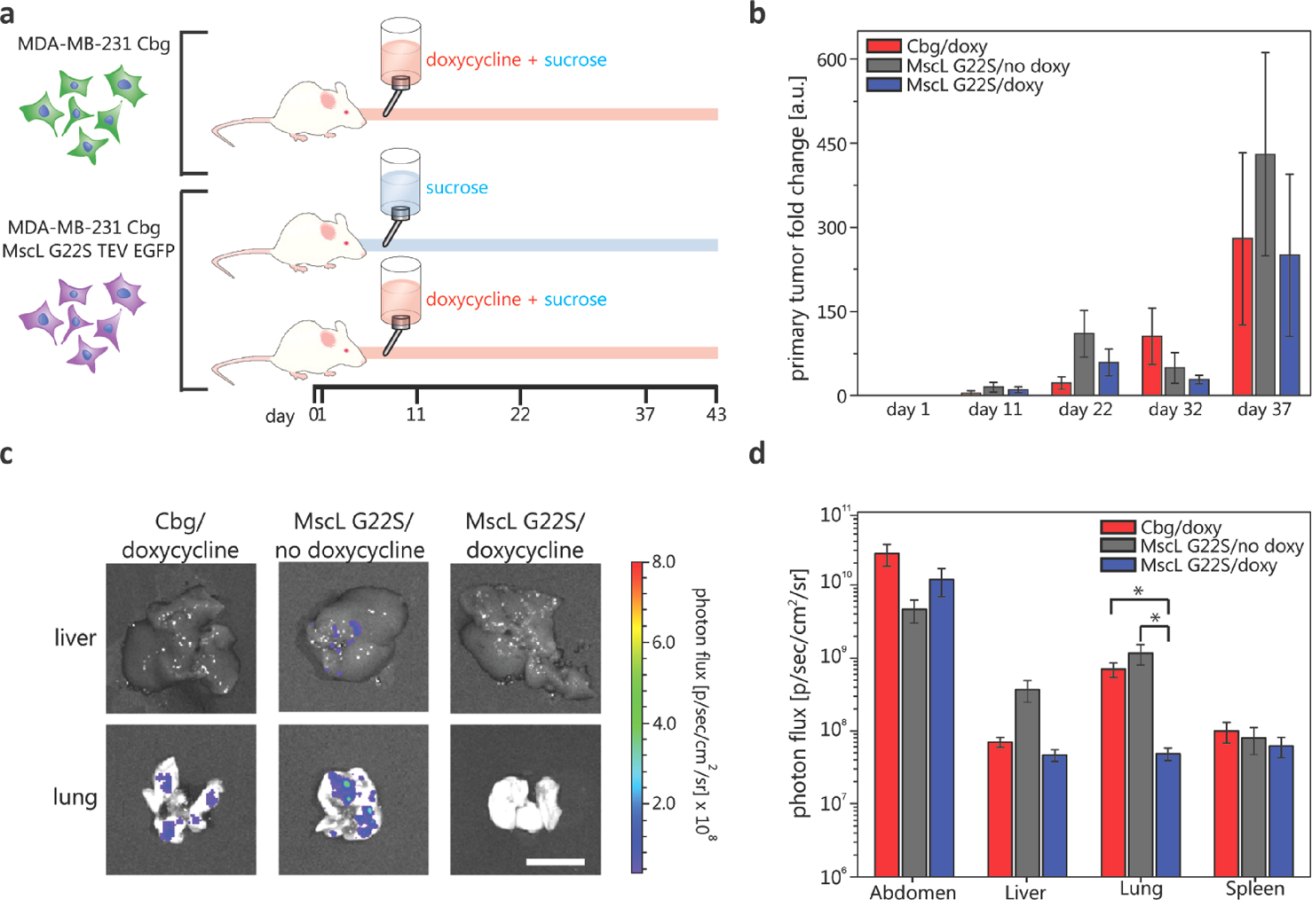
*In vivo* experiment for determining MscL's effect on cancer cell metastasis. (a) Cartoon description of *in vivo* experiments. MDA-MB-231 cells with doxycycline inducible expression of MscL G22S and constitutive luciferase expression and MDA cells with constitutive luciferase-only were injected under the mammary fat pad of immunodeficient mice on day 0. Three cohorts of mice were then studied: negative control group (1) mice with MDA-MB-231 MscL G22S luciferase cells with sucrose feed (n = 4), (2) mice with MDA-MB-231 luciferase only cells with doxycycline and sucrose feed (n = 5), and experimental group (3) mice with MDA-MB-231 MscL G22S luciferase cells with doxycycline and sucrose feed (n = 5). (b) Mean primary tumor size fold change at the site of initial injections as determined using bioluminescence imaging of mice on different days. Error bars represent the standard error of the mean. Differences in the total area-under-the-curve for bioluminescence do not differ among groups (p > 0.4). (c) Images of the extracted liver and lung with luminesce signal false coloring and the corresponding photon flux scale from a mouse of each cohort on day 43 relating to metastatic cancer cells at these secondary sites. Scale bar = 1 cm. The logarithmic plot of the average luminescence signal, the result of metastatic cancer cells, described as photon flux for various organs of each cohort. Error bars represent the standard error of the mean. The vertical axis starts above the luminescence background signal at 5 × 10^6^ p/s cm^2^ sr. Two-tailed student *t*-test of log transformed data: *p ≤ 0.01.

To test our hypothesis that expression of MscL G22S disrupts cell migration and metastasis, we used MDA-MB-231 human breast cancer cells stably expressing a doxycycline-inducible MscL G22S construct. We injected MDA-MB-231 MscL G22S or control cells into mammary fat pads of NSG mice under three different experimental conditions: (1) MDA-MB-231 MscL G22S luciferase cells with sucrose water (control); (2) MDA-MB-231 luciferase only cells with doxycycline in sucrose water (control); and (3) MDA-MB-231 MscL G22S luciferase cells with doxycycline in sucrose water (experimental)^10,23^ [Fig. 1(a) and Fig. 1 in the supplementary material]. Cohorts 1 and 2 were used to assess the effects of the noninduced MscL G22S construct and doxycycline, respectively. Bioluminescence imaging showed no difference in numbers of cancer cells in orthotopic tumors over ∼5 weeks, establishing that MscL G22S did not affect local tumor growth in the mammary fat pad [Fig. 1(b)]. When we euthanized mice for humane endpoints of tumor size, we used *ex vivo* bioluminescence imaging to detect and quantify metastases [Fig. 1(c)]. The most notable finding is the reduced metastasis in the lung for cohort 3 with induction of MscL G22S relative to cohorts 1 and 2 [Fig. 1(d)], while no other organs had significant differences. This result indicates that MscL G22S expression in metastatic breast cancer cells can impair metastasis. However, whether the effect is due to specific disruption of cell migration in narrow 3D confinements cannot be discerned. To examine the effects of MscL G22S in confined spaces, we next studied cell migration using an *in vitro* microfluidic system that mimics narrow cross-sections we suspect, leading to MscL's ability to disrupt migration and metastasis.

### Stable and functional expression of bacterial MscL in MDA-MB-231

**FIG. 2.**
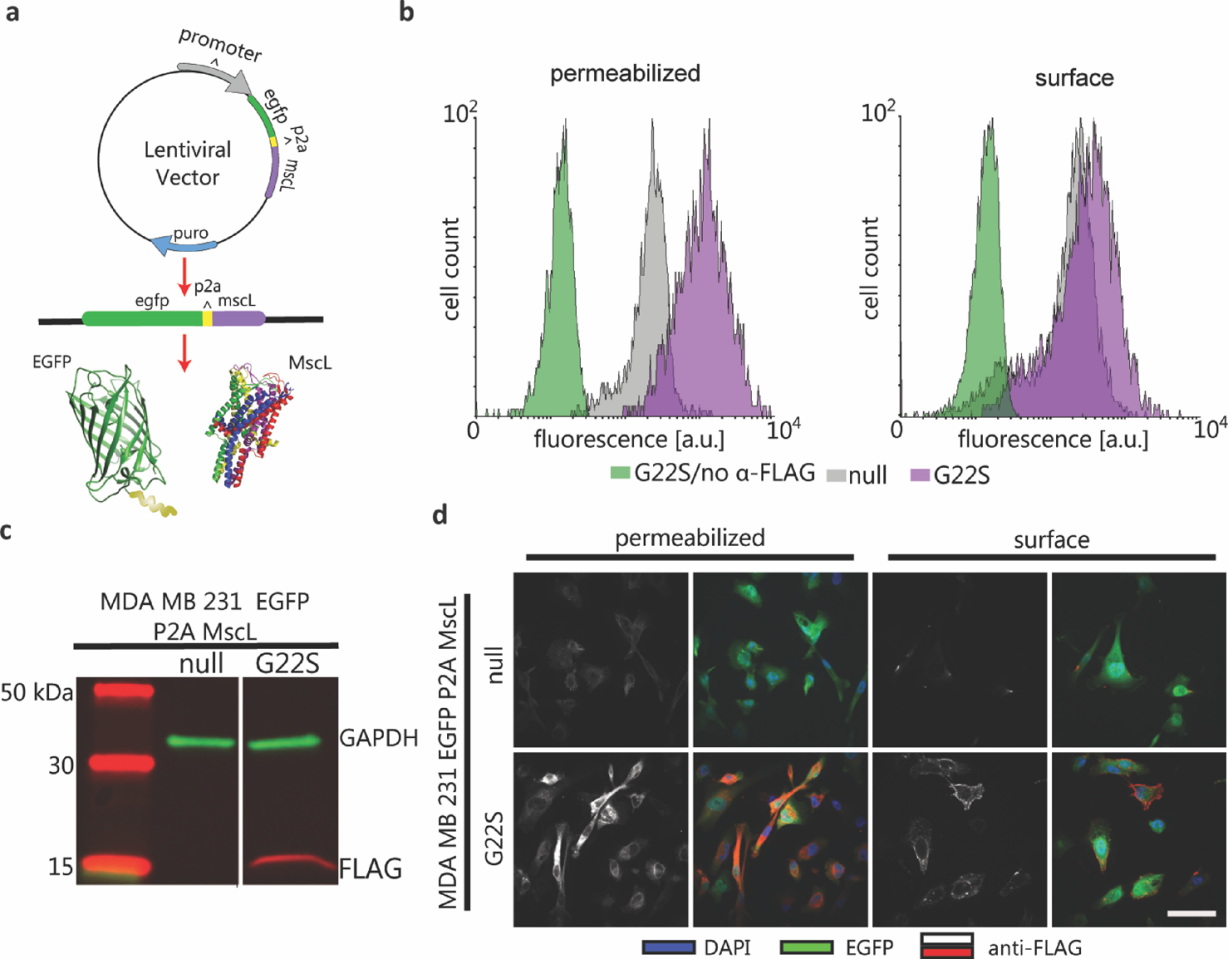
Lentiviral expression system for constitutive expression of MscL G22S in MDA-MB-231 cells. (a) A single lentivirus vector system for bicistronic expression of cytosolic EGFP and MscL from a single promoter. EGFP and MscL genes are encoded with a P2A linker sequence in between. Protein translation results in an incomplete peptide bond of the P2A linker's final amino acid, resulting in the expression of separate EGFP and MscL proteins. (b) Western blot analysis of transduced whole cells with a negative control vector, no MscL EGFP-only, and experimental cells, EGFP-P2A-MscL G22S with the periplasmic FLAG-tag. GAPDH was used as a housekeeping protein. (c) Flow cytometry fluorescence analysis using anti-FLAG Alexa Fluor^®^ 647 of methanol fixed and permeabilized cells (left) and PFA fixed cells for surface analysis (right). Negative controls were EGFP-P2A-MscL G22S cells with no anti-FLAG and no MscL EGFP-only cells with anti-FLAG, and experimental cells were EGFP-P2A-MscL G22S with anti-FLAG. (d) Immunostaining of FLAG for no MscL EGFP-only cells (top) and EGFP-P2A-MscL G22S with FLAG-tag (bottom) with methanol permeabilization and fixation (2 leftmost panels) and PFA fixed cells for surface analysis (2 rightmost panels). DAPI was used to label cell nuclei. Scale bar = 25 *μ*m.

To eliminate the need for doxycycline induction and luciferase expression, we engineered constitutively expressed MscL G22S and EGFP in MDA-MB-231 cells for subsequent *in vitro* studies. We fused a FLAG epitope tag to MscL G22S to facilitate immunodetection of MscL. Control cells stably expressed EGFP alone (also referred to as no MscL, EGFP-only) [Fig. 2(a)]. Whole-cell Western blot analysis using an anti-FLAG antibody showed robust expression of bacterial MscL G22S [Fig. 2(b)]. In previous studies of MscL expressed in mammalian cells, MscL localized to the plasma membrane and multiple intracellular, membrane-bound organelles.^11,12^ We confirmed this pattern of expression by flow cytometry on intact and permeabilized cells and by immunofluorescence staining [Figs. 2(c) and 2(d) and Fig. 2 in the supplementary material]. In both cases, we identified MscL on both plasma and intracellular membranes, verifying patterns of expression reported in other types of mammalian cells.

**FIG. 3.**
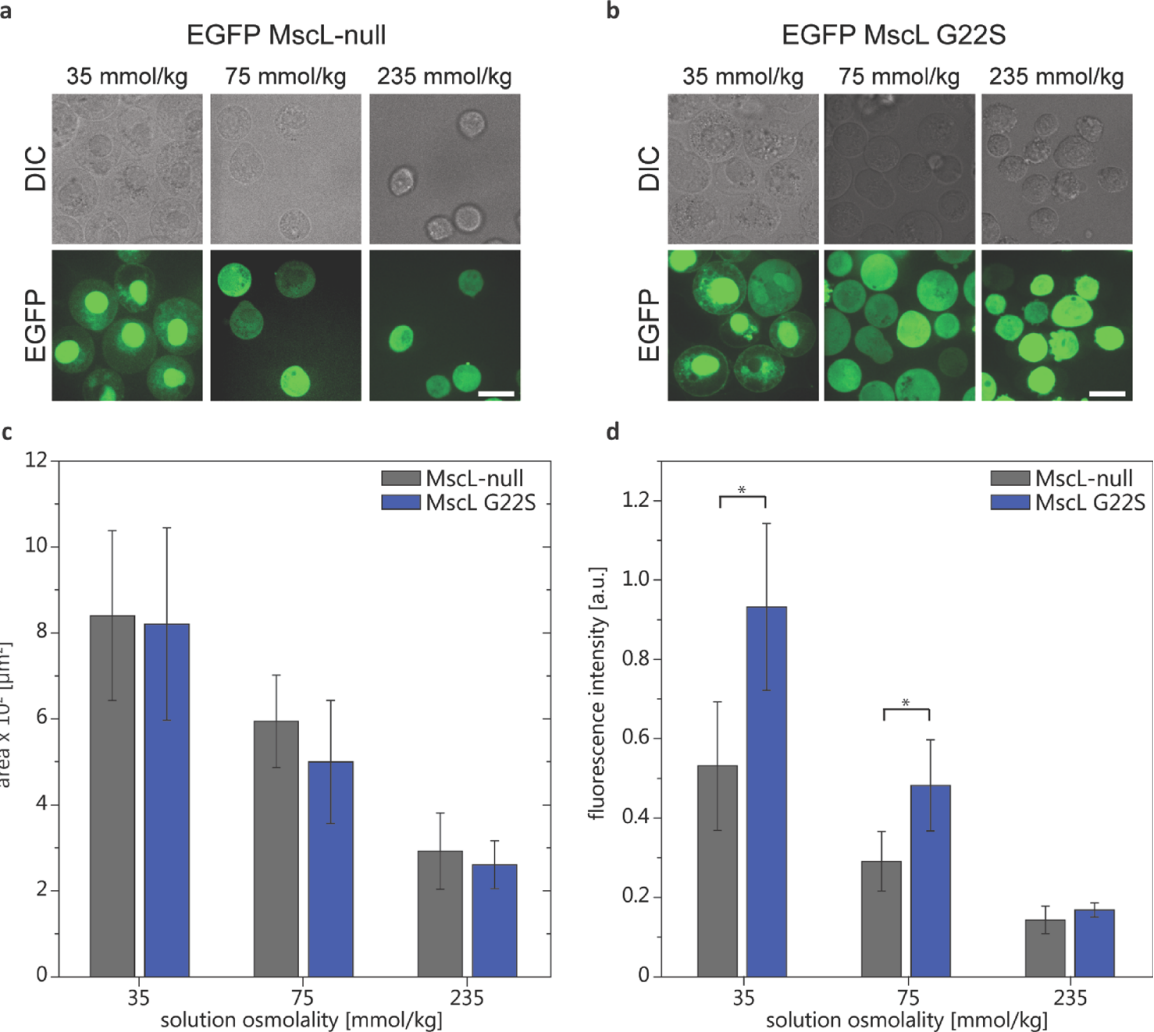
Osmotic downshock functional assay for MscL in MDA-MB-231 cells. Phase and fluorescence confocal images of cells suspended in solutions of varying osmolality (e.g., 235 mmol/kg—isotonic condition and 35 mmol/kg—hypotonic condition) with 100 *μ*M of impermeable PI after 3 min of (a) MDA-MB-231 MscL G22S cells and (b) MDA-MB-231 no MscL cells. (c) Comparison of the average area of the cell midplane cross-section for different osmotic conditions for no MscL and MscL G22S cells. The inset shows the comparison of the same cell type average area for different osmotic conditions (n_cells_ = 26–80). (d) Average fluorescence intensity of PI uptake for cells in suspension at varying osmolality (n_cells_ ≈ 10^5^, n_exp_ = 10) for 6 min. Two-tailed student t-test: *p ≤ 0.05.

To establish that MscL acts as a mechanosensitive channel in MDA-MB-231 cells, we subjected these cells to hypo-osmotic shock and quantified the uptake of an impermeable nucleic acid dye, propidium iodide (PI).^11^ This assay capitalizes on the known function of MscL in bacteria to prevent cell lysis under hypo-osmotic conditions by sensing increased membrane tension and allowing equilibration of osmolytes across the bacterial cell inner membrane.^24^ Hypo-osmotic shock increased sizes of both WT and MscL G22S MDA-MB-231 cells [Figs. 3(a)–3(c)]. The large size of MscL's open pore, ∼30 Å, allows free passage of osmolytes ≤10 kDa,^12,25^ and so, opening of MscL readily permits the intracellular entry of PI. Intracellular fluorescence from PI increased as the solution osmolality decreased [Fig. 3(d)]. MDA-MB-231 MscL G22S cells accumulated significantly more PI than control cells when incubated in 75 mmol/kg and 35 mmol/kg conditions. These results demonstrate that MscL G22S can be expressed and mechanically gated in metastatic breast cancer MDA-MB-231 cells.

### *In vitro* microfluidic migration device with narrow 3D confinements

**FIG. 4.**
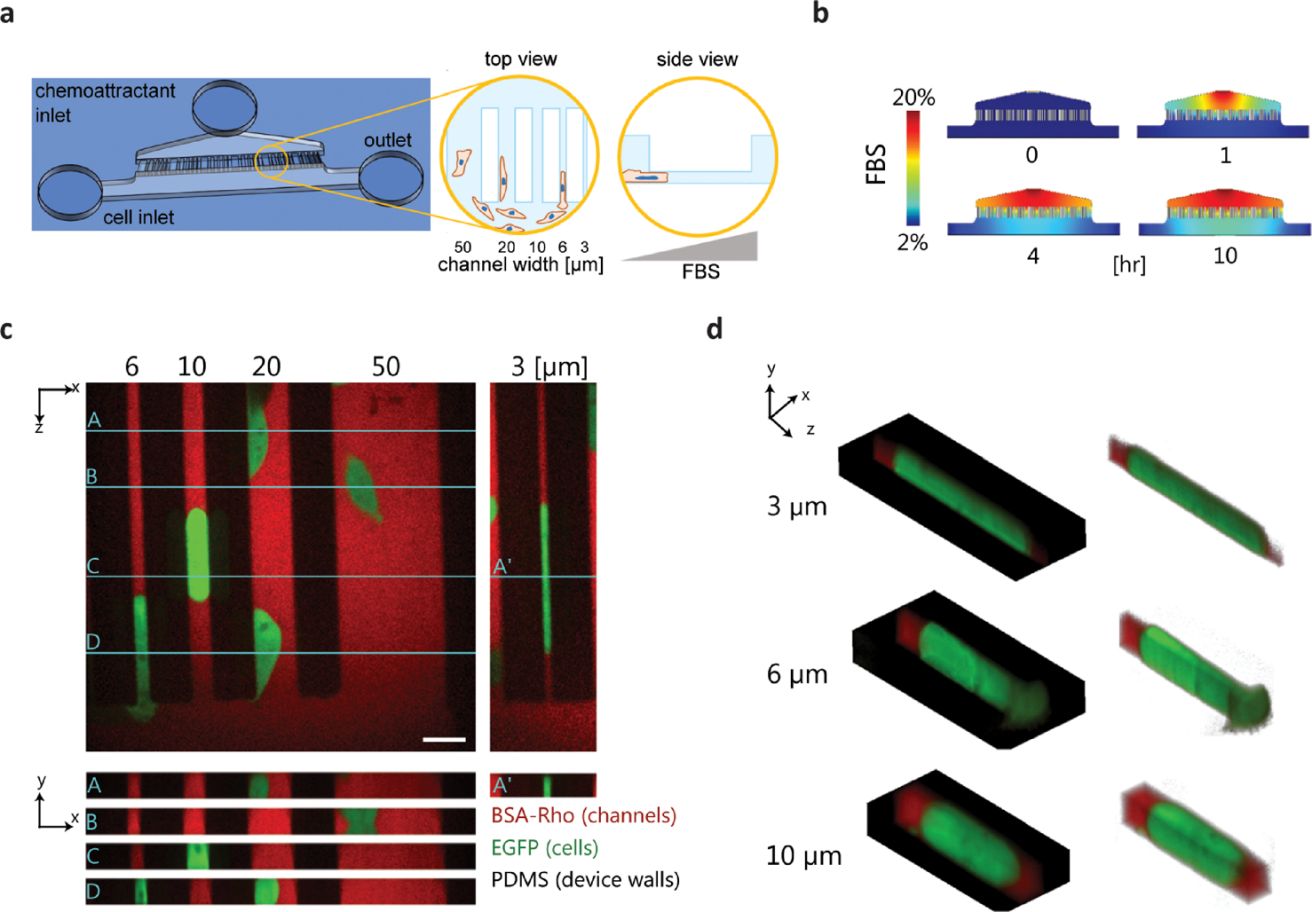
Microfluidic platform for studying cancer cell migration across 2D channels and narrow 3D constrictions. (a) Cartoon of the PDMS microfluidic migration device. Cells were added to the device via a cell inlet and flow into the bottom section of the device, adhered to the glass substrate, and migrated across channels of various sizes in response to an FBS chemoattractant gradient as shown in the zoomed-in views. (b) Images of COMSOL multi-physics simulation of the FBS gradient in the microfluidic device at different time points. (c) Fluorescence images showing orthogonal views, *y*-*x* and *z*-*x*, of EGFP expressing cells in the microfluidic device channels. The *z*-*x* cross-sections of the labeled, turquoise lines on the *y*-*x* view are shown for cells in all channel widths. The PDMS/device walls appear black in the images, and the channels were filled with media containing BSA-rhodamine. Scale bar = 20 *μ*m. (d) Isometric view of the cells in the narrow 3D constriction channels.

To test how MscL impacts cell migration in confined microchannels, we designed a microfluidic device with channels of varying cross-sections for 2D, planar (channel widths: 20 and 50 *μ*m) and narrow, 3D confined migration (channel widths: 3, 6, and 10 *μ*m) [Fig. 4(a)]. Using COMSOL to simulate the gradient dynamic, we determined that addition of serum to one channel established a gradient across all migration channels within one hour. The chemotactic gradient remained at equilibrium for >10 h [Fig. 4(b)]. Confocal imaging showed that cells entered different sized channels. Cells in the larger, 2D channels were not in contact with all of the channel walls (only top and bottom), while cells in the narrower confinements appeared to completely “plug” the channels [Fig. 4(c)]. Isometric volume views demonstrate the extent of cell deformation and conformation to the channel shape [Fig. 4(d)]. Cancer cell migration under this type of confinement has been shown to require the osmotic engine migration mechanism.^10,23^

### Functional expression of MscL G22S is not sufficient to disrupt cell migration in narrow 3D confinements

**FIG. 5.**
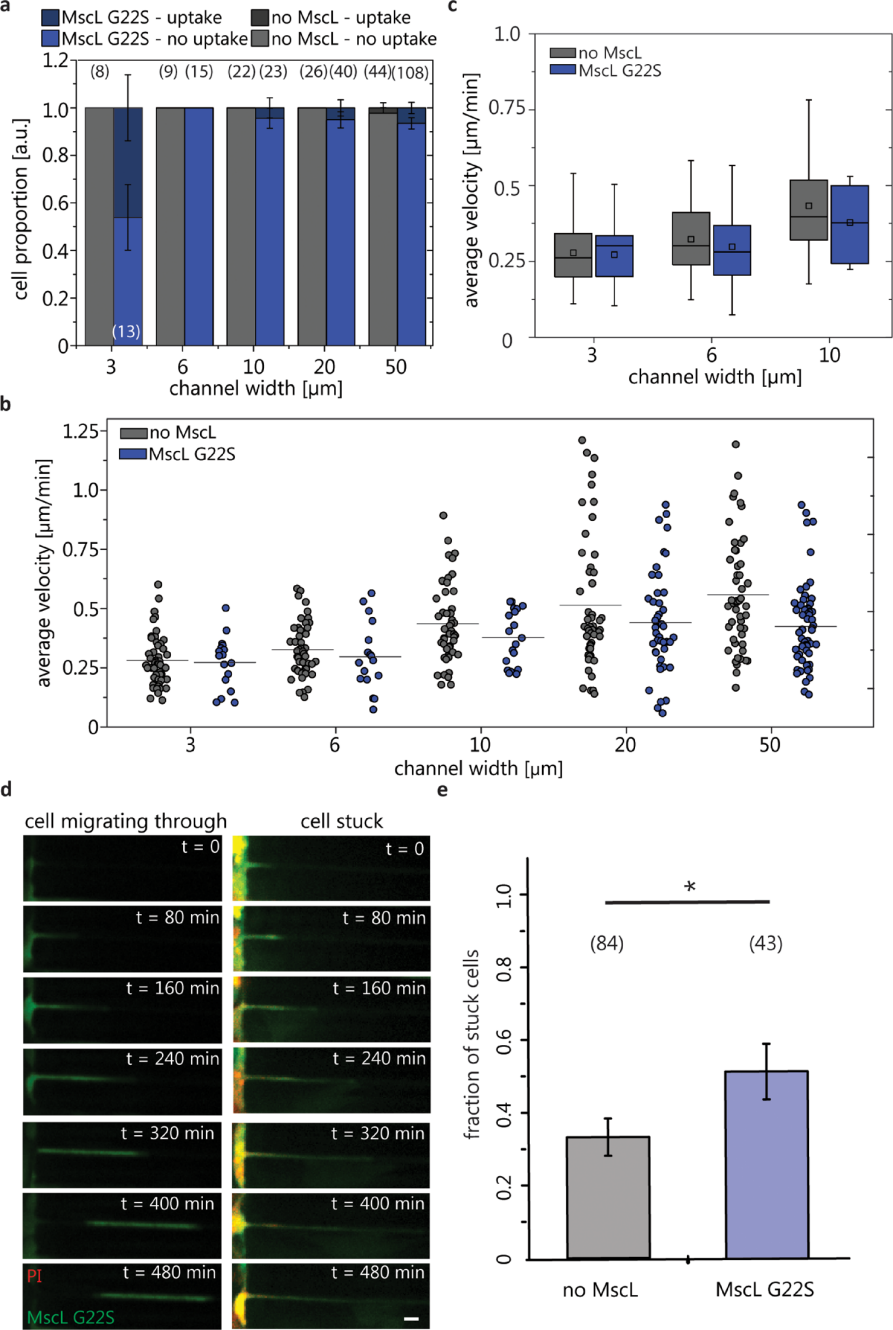
Migration of cancer cells in a microfluidic device. (a) Proportion of cells exhibiting PI uptake while entering or migrating across channels for different channel widths (the number of cells are denoted in parenthesis; error bars denote the standard error of proportion). (b) Average velocity of MDA-MB-231 no MscL and MDA-MB-231 MscL G22S cells in different sized channels of the microfluidic device. Circles represent the average migration of individual cells; black bars are the average velocity for all cells for the given channel width. 2-way ANOVA shows that the interaction between the cell type and the channel width is not significant, and so, the effect on cell migration in different channel widths does not depend on MscL expression. (c) Box plots of average cell migration for no MscL and MscL G22S cells in the confined channels. (d) Example of time-lapse fluorescence images of an MDA-MB-231 MscL G22S cell that migrated (left) or was stuck (right) in 3 *μ*m channels. The scale bar is 10 *μ*m. (e) Fractions of stuck MDA-MB-231 cells without and with MscL G22S. Error bars represent the standard error of proportion. The total number of cells is denoted in parenthesis. *p < 0.05.

Our *in vivo* mouse experiments showed that introduction of MscL G22S into MDA-MB-231 cells impaired metastasis to lungs. Using the *in vitro* microfluidic migration device, we tested to what extent this outcome resulted from MscL disrupting cell migration in narrow confinements. The phase imaging and fluorescence time-lapse imaging of the cancer cells were used to determine the influence of channel size and MscL G22S expression on cell migration. Cells migrated across the device in the direction of the chemoattractant gradient for ∼10–12 h (Fig. 3 in the supplementary material), and their change in the position was manually tracked (Movie 1 in the supplementary material). The actin cytoskeleton morphology was strikingly different when cells were in 3 *μ*m channels with actin caps at both ends instead of the presence of stress fibers in cells in 20 *μ*m or greater channels, as also previously observed by others^23^ (Fig. 4 in the supplementary material). The cell PI dye uptake was used to quantify MscL G22S activation in cells migrating in the device. We found a low proportion, 0%–6%, of MscL G22S cells taking up dye when entering channels of 6, 10, 20, and 50 *μ*m widths [Fig. 5(a)]. In contrast, a larger proportion, ∼46%, of MscL G22S cells showed the PI uptake when entering the narrowest channel of 3 *μ*m width. We found that the average cell velocity for the population of cells in the different sized channels decreased with the narrower channel width for both no MscL and MscL G22S cells [Fig. 5(b)]. For control MDA-MB-231 cells, we measured greater intercellular variations in velocities for cells in 10, 20, and 50 *μ*m width channels, following a broader distribution of single cell velocity measurements. MDA-MB-231 control cells in the 3 and 6 *μ*m width channels had narrower velocity distributions. The velocity measurement distributions for MDA-MB-231 MscL G22S cells were consistently narrower [Fig. 5(b)]. The comparison of the velocity of the no MscL and MscL G22S cells in 3, 6, and 10 *μ*m channel widths showed no statistically significant differences among the populations [Fig. 5(c)]. We found that the effect on cell migration in different channel widths did not depend on MscL expression. Interestingly, of the ∼46% of MscL G22S expressing cells that showed MscL activation by the PI uptake and entered the 3 *μ*m wide channels, we observed that some cells were “stuck” at the entrance of 3 *μ*m microchannels without fully entering the microchannels and did not migrate across [Fig. 5(d)]. Based on this observation, we quantified the fractions of cells that were stuck (cells trying to make it into the channel and either not entered all the way or backed out) or migrated through 3 *μ*m channels from the migration videos. The fraction of stuck cells was significantly higher for MscL G22S expressing cells than cells without MscL [Fig. 5(e)]. These results suggest that MscL expression and activation impact the cell entry into 3D confined channels, but those cells that were able to enter microchannels did not migrate differently.

## DISCUSSION

In the present work, we show that expression of MscL G22S in human breast cancer cells impaired lung metastasis in mice. However, when cell migration *in vitro* was examined using microfluidic channels with varying 3D confinements, we did not observe changes in cell migration velocity between control and MscL G22S cells. MscL activation was found for ∼46% of cells that “entered” or migrated across the 3 *μ*m width channel where the osmotic engine migration mode would be activated.^10^ Given this large proportion of activated cells, if our hypothesis was correct, there would have been a more pronounced effect on cell migration, where we presume that MscL activation would abrogate polarized water and ion fluxes necessary for the osmotic engine to function. This may indicate that activation of MscL as a result of migration across narrow, 3D confinements was not sufficient to hinder cell migration *in vitro*. Interestingly, we found that a large fraction of MscL activated cells were stuck at the entrance of 3 *μ*m microchannels and did not fully enter the microchannels. By comparison, those cells that migrated in 3 *μ*m microchannels did not activate MscL. In agreement with this observation, there were significantly less MscL G22S cells that migrated into 3 *μ*m microchannels [see Figs. 5(b) and 5(c)]. Together, this would explain why we found the same migration velocity for no MscL and MscL G22S cells. This finding suggests that MscL G22S cells have impaired abilities to enter confined spaces, and this is a self-selecting process. Since most MscL G22S expressing cells that migrated into 3 *μ*m microchannels did not have PI uptake, we would not be able to test whether MscL activation can alter 3D confined migration.

It is unclear why functional expression of MscL G22S decreased metastasis only to the lung. Breast cancer has preferential metastasis to the bone, lung, liver, and brain. The reasons for this have been linked to genetic factors in breast cancer cells, differences in constituents of various tissues, and formation of pre-metastatic niches. Ongoing research also shows that physical factors regulate organ-specific tropism of metastasis.^2,3,26,27^ Each organ has very distinct and different tissue architectures, mechanical properties, and compositions. For example, lung vasculature maintains tight junctions to restrict permeability, while both the liver and bone have fenestrated sinusoids that facilitate the access of cells and solutes.^28–30^ This and additional geometric and mechanical differences in lung versus other potential metastatic sites may account for selective effects of MscL on metastases to this organ, but further studies will be required to determine mechanisms.

While several approaches based on mechanical actuation using force-field gradients have emerged as complementary technologies to manipulate cell signaling,^31^ microfluidics has become a powerful tool for directly manipulating the physical microenvironment. A number of studies have investigated cell migration mechanisms in 3D and in confined geometries using microfluidics,^32–35^ but how this is related to *in vivo* scenarios remains to be seen, given the complex patterns of metastasis. Our work on “repurposing” bacterial mechanosensitive channels in mammalian cells introduced novel mechanotransduction capabilities by introducing exogenous mechanosensory components. This approach opens new areas of inquiry in mechanobiology and may inspire new ways of engineering cellular mechanosensitivity.

## METHODS

### Cells

MDA-MB-231 human breast cancer cells were cultured and maintained in RPMI 1640 supplemented with 10% fetal bovine serum, 10 000 units/mL of penicillin, 10 000 *μ*g/ml of streptomycin, and 25 *μ*g/ml of amphotericin B. Human embryonic kidney (HEK) 293T cells (ATCC) were cultured in Dulbecco's Modified Eagle Media (DMEM) with 10% fetal bovine serum, 1% penicillin, streptomycin, and 2 mM glutamine added. Both cell lines were cultured in an incubator set to 5% CO_2_ and 37 °C.

### Lentiviruses

A gene block with the sequence for MscL G22S fused to enhanced Green Fluorescence Protein (EGFP) via a consensus cleavage sequence for a Tobacco Etch Virus (TEV) protease (IDT) was ordered, and the DNA was inserted into the EcoRI and BamHI sites of the pLVX TetOne puromycin lentiviral vector (Takara). A gene block with optimized human codons for the TEV protease (IDT) was ordered, and this DNA was cloned into XbaI sites of lentiviral vector FUeqFP650. We generated lentiviruses in HEK 293T cells as described.^36^

### Orthotopic mouse model of breast cancer

MDA-MB-231 cells with doxycycline inducible expression of MscL G22S and constitutive click beetle green luciferase expression (231-MscL-CBG) and MDA-MB-231 cells with constitutive click beetle green luciferase-only (231-CBG) were injected bilaterally into the 4th inguinal mammary fat pads of 6–10-week-old female NSG mice (Jackson Laboratory) on day 0. Three cohorts of mice were then studied: (1) 231-MscL-CBG mice treated with 1% sucrose in drinking water (n = 4); (2) 231-CBG mice with water containing 2 mg/ml doxycycline and 1% sucrose (n = 5); and (3) experimental group mice with 231-MscL-CBG cells and with water containing both doxycycline and sucrose as for group 2 (n = 5). The primary tumor size was tracked at the site of injection for each mouse via bioluminescence imaging (IVIS Spectrum, Perkin-Elmer). At day 43, the mice were euthanized and bioluminescence images of the liver, lung, abdomen, and spleen were taken to quantify metastases. The statistical significance testing of primary tumor growth data consisted of two-tailed student t-tests amongst all groups for each day. The statistical significance testing of the photon flux, metastasis data consisted in taking the log transform of all data points and performing a two-tailed *t*-test.

### Stable expression of MscL in MDA-MB-231 cells

DNA constructs for the *Escherichia coli* MscL WT were kindly provided by Boris Martinac (Victor Chang Cardiac Research Institute, Darlinghurst, Australia). The MscL WT construct was mutated to produce MscL G22S using a Q5^®^ Site Direct Mutagenesis Kit (New England Biolabs, Ipswich, MA) and then subcloned into a lentiviral vector pLVX Puro (Takara Bio USA, Inc, Mountain View, CA). Lentivirus from constructs encoding only EGFP (negative control, EGFP-only) and EGFP-P2A-MscL G22S with the FLAG epitope tag inserted just after MscL residue I68^37^ were harvested from HEK 293T cells. Metastatic breast cancer cells, MDA-MB-231, were transduced with either virus.

### Immunofluorescence and flow cytometry

For immunofluorescence imaging and flow cytometry analysis, MDA–MB-231 cells were fixed and permeabilized using methanol or fixed using 2% paraformaldehyde (PFA) for surface labeling with anti-FLAG iFluor 647 antibodies (Genscript, Piscataway, NJ). The analysis of infrared Western blots was conducted using a LI-COR Odyssey Sa system (Lincoln, NE), and flow cytometry analysis was performed on a Millipore Sigma Guava^®^ easyCyte flow cytometer (Burlington, MA).

### MscL osmotic shock functional assay

MDA-MB-231 cells were suspended in solutions of varying osmolality (measured using a vapor pressure osmometer) in base media diluted with dI water containing 100 *μ*M propidium iodide (PI) (Sigma-Aldrich, St. Louis, MO). PI is impermeable to the plasma membrane and labels nucleic acids upon entering the cells. We validated the use of PI in our previous work^11^ and showed that activation of MscL alone did not lead to cell death by post-labeling with PI. Prior to incubation, all cells were detached from culture dishes using 5 mM EDTA and counted using a Millipore Scepter™ to assure that each bulk sample contained an equal number of cells of ∼10^5^. The sample fluorescence intensity due to PI uptake by the cells was measured using a Biotek Synergy H1 plate reader (Winooski, VT). The background fluorescence intensity was subtracted from data, and the values were normalized to the number of cells per bulk sample. The fluorescence imaging of the samples was also performed on a Biotek Cytation 5 imaging plate reader, and image processing was done using ImageJ (http://rsb.info.nih.gov/ij/).

### *In vitro* microfluidic migration device

The microfluidic device including 2D migration channels (cross-section: 50 *μ*m × 10 *μ*m and 20 *μ*m × 10 *μ*m; vertically constrained) and narrow 3D constriction channels (cross-section: 10 *μ*m × 10 *μ*m, 6 *μ*m × 10, and 3 *μ*m × 10 *μ*m; both vertically and horizontally constrained) was designed to be similar to previous migration devices.^10,23^ The dimensions of the cell seeding channel and the fetal bovine serum (FBS) channel have areas of 0.0040 and 0.0048 mm^2^, respectively. Both of these channels have a height of 61 *μ*m (measured using a profilometer). Compared to the height of 10 *μ*m of the microchannels, the cell seeding and FBS channels act as an infinite sink and source, respectively. The device was designed to allow the chemoattractant gradient of fetal bovine serum (FBS) to be quickly established (∼1 h) across all channels and such that the gradient remained uniform and steady over the course of ∼12 h, as verified through COMSOL Multiphysics (Burlington, MA) simulations. Soft lithography was used to fabricate the device with polydimethylsiloxane (PDMS) (Dow Chemical, Midland, MI). The device design layout was drawn using AutoCAD and printed onto a lithographic mask by CAD/Art Services (Bandon, OR) and a Chrome mask by Photo Science, Inc. (Torrance, CA). The mask pattern was transferred onto SU-8 on a silicon wafer to produce the device mold for forming the PDMS casting. After punching inlets and outlets, the PDMS cast was then permanently bonded to a glass slide or coverslip following oxygen plasma treatment.

### *In vitro* migration experiments

Assembled microfluidic migration devices were treated with 50 *μ*g/ml of human fibronectin (Sigma Aldrich, St. Louis, MO) in 1× phosphate buffer saline (PBS). The devices were then incubated for 1 h at room temperature. After incubation, the devices were rinsed with 1× PBS and filled with phenol red-free RPMI 1640 media supplemented with 2% FBS and stored at 37 °C. MDA-MB-231 cells were then detached from cell culture dishes using 5 mM EDTA and resuspended in phenol red-free RPMI 1640 media supplemented with 2% FBS at a concentration of 1 × 10^6^ cells/ml. Immediately after this, the cells were slowly pipetted into the cell inlet of the microfluidic migration device. The gravity-driven flow, as a result of differential hydrostatic pressure in the device inlets and outlets, pushed the cells near the opening of the channels. Once a sufficient number of cells were present near the entrance of the device channels, the gravity flow was removed by equalizing the height of the media in all inlets and outlets. The cells were incubated in the device for 30 min at 37 °C and 5% CO_2_ to allow them to adhere to the device bottom surface. The media was then gently removed from the chemoattractant inlet and replaced with the media supplemented with 20% FBS. Migration devices were then mounted onto an Olympus spinning disk confocal microscope (Yokogawa CSU-X10). Time-lapse, Δt = 12 min, differential interference contrast (DIC), and fluorescence images of cells in the devices were taken for 8–12 h on an Andor EMCCD camera using image acquisition software Metamorph.

For determining percentages of activated and non-activated cells, cells were seeded in the migration device and allowed to adhere and migrate for 8 h. PI was added to all the device inlets for an hour, and then, images were collected. PI uptake was a binary measurement (i.e., uptake or no uptake) determined by an intensity threshold for PI fluorescence that was set using the MDA-mB-231 no MscL cells as control images.

For determining the fraction of stuck *vs.* migrating cell and the correlation with MscL activation in the 3 *μ*m channel, MDA-MB-231 cells expressing MscL G22S were seeded in the device and PI was added right after. Imagining began 1 h after cell seeding and PI addition, and the cells were tracked in both fluorescence channels over 9 h.

### Migration tracking and data analysis

Time-lapse movies of cell migration in devices were first saved and organized using ImageJ software. For each movie, frame drift correction and manual cell position tracking were performed using a MATLAB CellTracker.^38^ All cells were binned into groups based on the width of the channel the cell travelled across. Cells were only tracked when fully inside of the channel and were tracked exclusively at the leading end. The average velocity for each cell was then calculated, and the velocity distribution for each channel width was displayed using boxplots. The statistical significance testing of migration data consisted of a two-way analysis of variance (ANOVA) analysis with the Tukey post-hoc test. The standard error of proportion was calculated for data based on counting. The 95% confidence *z*-value of 1.96 was used to determine the statistical significance based on the difference between sample means ± *z*× the standard error for difference.

Recombinant DNA work has been approved by Institutional Biosafety Committee (IBCA000000005_AR01), and animal work has been approved by the UM Institutional Animal Care and Use Committee (6795). No human subjects were involved in the present study.

## SUPPLEMENTARY MATERIAL

See supplementary material for four supplementary figures and one supplementary movie.
